# Sequential co-assembly reduces computational resources and errors in metagenome-assembled genomes

**DOI:** 10.1016/j.crmeth.2025.101005

**Published:** 2025-03-17

**Authors:** Hannah M. Lynn, Jeffrey I. Gordon

**Affiliations:** 1 Edison Family Center for Genome Sciences and Systems Biology, Washington University School of Medicine, St. Louis, MO 63110, USA; 2 Newman Center for Gut Microbiome and Nutrition Research, Washington University School of Medicine, St. Louis, MO 63110, USA

**Keywords:** metagenome-assembled genomes, shotgun DNA sequencing reads, sequential co-assembly, assembly time, single-node computing tools, misassembly errors, memory requirements, MEGAHIT, Bowtie

## Abstract

Generating metagenome-assembled genomes from DNA shotgun sequencing datasets can demand considerable computational resources. Here, we describe a sequential co-assembly method that reduces the assembly of duplicate reads through successive application of single-node computing tools for read assembly and mapping. Using a simulated mouse microbiome DNA shotgun sequencing dataset, we demonstrated that this approach shortens assembly time, uses less memory than traditional co-assembly, and produces significantly fewer assembly errors. Applying sequential co-assembly to shotgun sequencing reads from (1) a longitudinal study of gut microbiomes from undernourished Bangladeshi children and (2) a 2.3-terabyte dataset generated from gnotobiotic mice colonized with pooled microbiomes from these children that was too large to be handled by a traditional co-assembly approach also demonstrated significant reductions in assembly time and memory requirements. These results suggest that this approach should be useful in resource-constrained settings, including in low- and middle-income countries.

## Highlights

• Sequential co-assembly reduces redundant sequence assembly in metagenomics datasets
• Sequential co-assembly uses less memory and is faster than traditional co-assembly
• Sequential co-assembly reduces assembly errors in simulated sequencing datasets
• This approach can also assemble datasets that are too large for traditional co-assembly

## Motivation

Co-assembly of DNA shotgun sequencing reads from multiple microbial community samples into metagenome-assembled genomes is an important approach for reconstructing bacterial genomes from longitudinal or cross-sectional microbiome studies but is time and memory intensive. Here, we present an approach that involves sequential use of existing assembly and mapping programs run on single computing nodes to reduce assembly of redundant sequencing reads, co-assembly time, memory requirements, and assembly errors compared to traditional one-step co-assembly. A goal in developing this approach is to facilitate more advanced metagenomic analyses in computational-resource-limited settings.

## Graphical abstract

**Figure:**
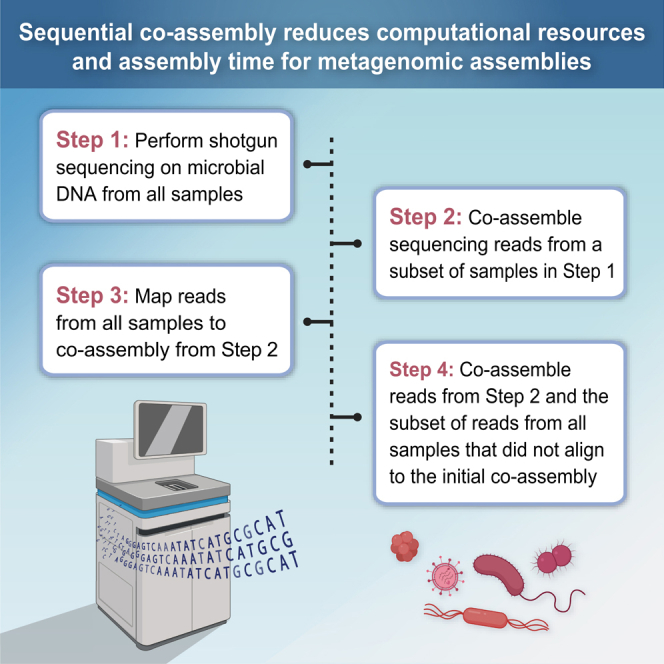

## Teaser

Assembly of shotgun sequencing reads into metagenome-assembled genomes is computationally intensive. Lynn and Gordon have developed “sequential co-assembly,” an approach that reduces the number of redundant sequences in metagenomics datasets. Compared to traditional methods, this allows assemblies to be completed in shorter times and with fewer computational resources.

## Introduction

Metagenome-assembled genomes (MAGs) generated from microbial community samples can be used in a variety of analytical applications including identifying organisms within a community that cannot be easily cultured,^1^ performing ecological analyses of genomes at strain resolution,^2^ and constructing reference genomes to which microbial gene-expression data can be mapped and profiled.^3^ The first step of MAG generation involves assembly of shotgun sequencing reads into contigs.^4^ Assembly can be accomplished using multiple approaches, including assembly of reads from individual samples, co-assembly from a group of related samples, or a combination of both methods^5^; the resulting contigs are subsequently grouped (“binned”) into MAGs.^6^

Regardless of the approach employed, assembly time and dataset size are influenced by several factors, including the “true” richness and evenness of the microbial community, the depth of sequencing, and the presence of informationally redundant sequencing reads. Redundant reads can arise for a variety of reasons, e.g., generation of PCR duplicates (identical sequencing reads) during preparation of DNA libraries for sequencing, sequencing a community to a depth greater than the minimum needed for assembly, or uneven sequencing coverage of genomes within a sample due to differences in their abundances.^7^ The following two examples highlight how sequencing depth and uneven genome coverage can greatly amplify the number of sequencing reads requiring assembly. If there is a community with three organisms whose abundances are equivalent and 10,000 sequencing reads/genome are required for complete assembly of each genome, the minimal number of reads required would be 30,000. Sequencing to a depth greater than 30,000 reads would yield redundant reads that would consume additional computational time and memory but would not yield additional information for the assembly. In a different scenario, for a community with three organisms where organism A has a relative abundance of 90% and organisms B and C each have relative abundances of 5%, obtaining at least 10,000 sequencing reads per genome would require sequencing to a depth of 200,000 reads (yielding 180,000 reads from organism A and 10,000 reads from both B and C). Removal of such duplicated reads has been shown to improve contig binning and decrease time and memory requirements ^8^; however, deduplication programs such as samtools^9^ and picard^10^ remove PCR duplicates but require a reference genome. Fastp^11^ and bbtools^12^ provide alternative deduplication tools that can be used with raw read files but can be computationally intensive.

These challenges, which continue to grow as sequencing costs decrease and dataset sizes increase, underscore the need to develop co-assembly strategies that are more memory and time efficient, particularly where computational capacity is limited (e.g., as is the case in many low- and middle-income countries where deep metagenomic surveys of human and environmental samples are often lacking despite the pressing need for them in global health and climate-change-related research).^13^ The importance of reducing memory requirements and assembly times for the metagenomics community was also emphasized in the most recent Critical Assessment of Metagenome Interpretation (CAMI) challenge, where these metrics were included as important endpoints for comparing assembly tools.^14^ Two notable, high-performing assemblers evaluated in the CAMI challenge included MEGAHIT and MetaHipMer. MEGAHIT employs succinct de Bruijn graphs to quickly assemble short reads into contigs but is limited to datasets with sizes that can be handled on a single-node computing system.^15^ In contrast, MetaHipMer has the capability to co-assemble datasets at terabyte scale within a matter of hours but does so by running the program across thousands of computing nodes on a supercomputer.^16^

Here, we describe an alternative “sequential co-assembly” strategy that utilizes the single-node processing of MEGAHIT and has the capacity for handling sequencing datasets of multiple terabytes. This method combines sequential application of multi-sample co-assemblies and read mapping to reduce the burden of duplicate, uninformative reads. Using three datasets, ranging in size from gigabytes to terabytes, we demonstrate its effectiveness in reducing co-assembly time, memory requirements, and assembly errors compared to a traditional one-step co-assembly.

## Results

### Applying sequential co-assembly to simulated mouse gut microbiome data with reference genomes

Sequential co-assembly was inspired by the idea of separating reads into different groups (i.e., those that align to a reference genome vs. those that do not) via mapping. This strategy is applied during read pre-processing steps of metagenomic pipelines to separate host and bacterial reads by mapping reads to the host organism’s genome^3^ and utilized by deduplication tools that rely on mapping of reads to a reference genome.^9^^,^^10^ Figure S1 compares the process of traditional one-step co-assembly to a sequential co-assembly approach. An example dataset is shown in Figure S1A, in which a mock community consists of four genomes (organisms), one of which is highly abundant in all samples, and the three others that have low abundances and are only present in a small subset of samples. A traditional co-assembly would involve combining reads from all samples and co-assembling the entire set of reads (Figure S1B). In contrast, sequential co-assembly begins with a co-assembly of reads of from a small subset of samples (Figure S1C, step 1). In this example, co-assembly of the subset of reads would yield the genome from the most abundant (red) organism. This genome would then serve as a “reference” genome for step 2, in which all reads from all samples (Figure S1C, step 2) are mapped to the “reference” genome in order to separate “uninformative” reads (those from the high-abundance organism whose genome contigs have already been assembled) from “informative” reads (reads from the low-abundance organisms whose genomes were not assembled in the initial co-assembly). The final step (Figure S1C, step 3) involves co-assembling reads from the initial subset of samples along with the remaining “informative” reads not aligning to the initial co-assembly in step 2. Ultimately both traditional and sequential co-assembly strategies assemble genomes from all four organisms in the community, but the latter approach reduces the number of duplicate reads requiring assembly compared to a traditional co-assembly.

**Figure 1:**
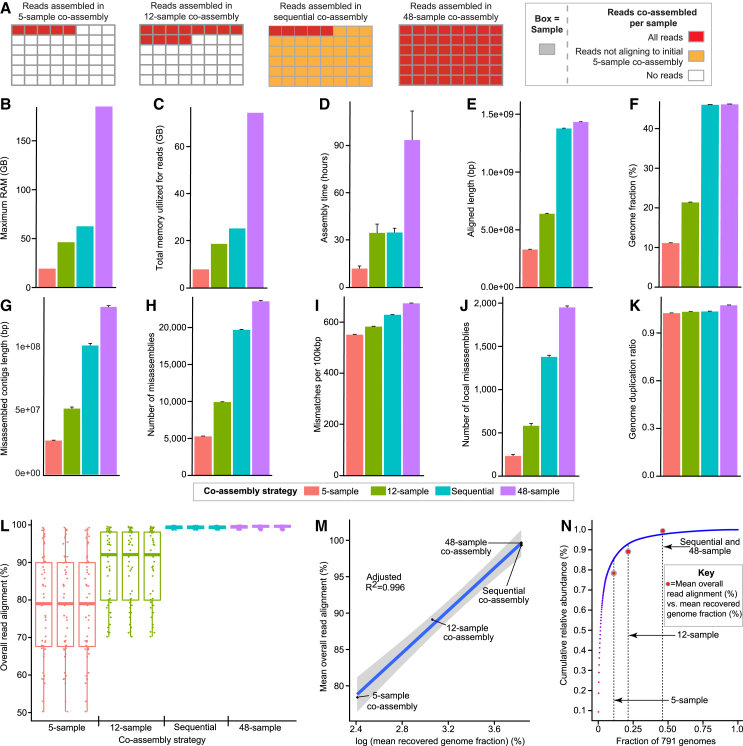
Comparing traditional and sequential co-assembly approaches for a simulated mouse gut microbiome dataset with reference genomes (A) Schematic of reads co-assembled for each of the four co-assembly strategies. (B–D) Barplots of MEGAHIT-reported metrics for each of the four co-assembly strategies, including (B) the maximum RAM (gigabytes, GB), (C) total memory required for reads (GB), and (D) assembly time (hours). (E and F) Barplots of MetaQUAST reported genome recovery metrics, including (E) aligned length to reference contigs (bp) and (F) recovered reference genome fraction (%). (G–K) Bar plots of MetaQUAST misassembly metrics comparing co-assembled contigs to the set of 791 reference genomes, including (G) misassembled contig length (bp), (H) total number of contig misassemblies, (I) mismatches per 100 kilobase pairs (kbp), (J) number of local miassemblies, and (K) genome duplication ratio. (L) Box-and-whisker plot capturing the distribution of overall read alignment (%) of reads from the 48 samples aligning to the 12 co-assemblies. Each point represents one of the 48 samples. (M) Scatterplot of the mean overall read alignment (%) vs. the log-transformed mean recovered genome fraction for each of the four co-assembly approaches. (N) Scatterplot of the cumulative relative abundance (%) vs. the fraction of the 791 genomes. The red dots represent superimposed values of the mean overall read alignment (%) vs. the mean recovered genome fraction for each of the four co-assembly strategies. The blue line in (M) shows the best fit line (linear model: mean overall read alignment [%] ∼ log(mean recovered genome fraction [%]). The gray area shows standard error around the best fit line. *n* = 3 assemblies per co-assembly strategy for (B)–(K). Error bars for barplots (D)–(K) show standard deviation. Error bars are not shown for (B) and (C) due to the same amount of memory being utilized by MEGAHIT across the three runs for each co-assembly strategy. Boxplots in (L) show minimum, first quartile, median, third quartile, and maximum values. See also Tables S1A–S1I.

We first applied the sequential co-assembly method to simulated shotgun sequencing reads from 48 samples in the “toy” mouse gut dataset from the second CAMI challenge.^17^ This dataset was simulated from a total of 791 microbial reference genomes from the NCBI RefSeq database. Each of the 48 samples has 5 GB of simulated Illumina HiSeq 2000 reads. The abundances of the genomes in the simulated samples are reflective of the 16S rRNA profiles of mouse gut microbiota samples. We performed four co-assemblies of varying scales to compare the sequential and traditional co-assembly approaches: three traditional co-assembly strategies included reads from (1) a subset of five samples, (2) a subset of 12 samples, (3) the entire set of 48 samples (see Figure 1A and STAR Methods), and a sequential co-assembly of reads from the five-sample subset, plus reads from all samples not aligning to the five-sample co-assembly (see Figure 1A and STAR Methods).

All co-assembly and read-mapping steps described in this paper were performed on a single-node server of a high-throughput computing cluster (STAR Methods). The four co-assembly strategies were performed with MEGAHIT^15^ (version 1.1.3, 8 central processing units [CPUs] per task) in triplicate to assess the “determinism” of assembly runs. The sequential co-assembly method utilized the initial five-sample co-assembly, plus two additional steps (numbered 2 and 3 in Figure S1C): (1) mapping reads from all 48 samples to the initial five-sample co-assembly using Bowtie 2^18^ (version 2.3.4.1) to remove reads aligning to the five-sample co-assembly, and (2) co-assembly of reads from all 48 samples that did not align to the five-sample co-assembly plus the full set of reads from the initial five samples (STAR Methods). The 12 sets of assembled contigs were compared to the 791 reference genomes using MetaQUAST^19^ (version 5.2.0, STAR Methods).

The maximum random access memory (RAM) utilized by MEGAHIT during assembly, the memory utilized by MEGAHIT for handling the reads, and the amount of time MEGAHIT took to perform the co-assemblies all increased with the number of co-assembled samples for the traditional co-assembly strategies: (1) five-sample co-assemblies utilized 19.28 GB and 7.74 GB, respectively, and had a co-assembly time of 11.71 ± 1.64 h; (2) 12-sample co-assemblies utilized 46.24 GB and 18.58 GB, respectively, and had a co-assembly time of 34.44 ± 5.57 h; and (3) 48-sample co-assemblies utilized 184.99 GB and 74.38 GB, respectively, and had a co-assembly time of 93.48 ± 18.60 h (mean ± SD) (Figures 1B–1D and Table S1A). Compared to the 48-sample co-assemblies, MEGAHIT utilized 66% less memory for maximum RAM and 66% less allocated memory for reads for the final assembly in the sequential co-assembly protocol (62.57 GB maximum RAM and 25.15 GB for reads) (i.e., step 3 in Figure S1C) (Figure 1B and Table S1A). Additionally, the average time required by MEGAHIT to assemble the reads for the sequential co-assembly was 63% less than that taken to complete the 48 sample co-assemblies (34.66 ± 2.69 h, mean ± SD) (Figure 1D and Table S1A). Furthermore, the total computational time for the sequential co-assembly (co-assembly of the five-sample co-assembly, mapping reads from all 48 samples to the five-sample co-assembly, plus the final co-assembly step) was less than the time to complete the traditional co-assembly approach for all 48 samples. The time needed to map reads from each of the 48 samples to each of the three five-sample co-assemblies (when performing individual sample mapping in parallel) ranged from 14.38 to 35.60 min (Table S1B). The cumulative mapping times (summation of all mapping times or mapping in series) ranged from 17.61 to 19.02 h, and total computational times from 46.96 h (parallel mapping) to 65.39 h (series mapping)—values that are 30%–50% less than the time required for the traditional 48-sample co-assembly approach.

The MetaQUAST reported aligned contig length (i.e., length of the contigs assembled by MEGAHIT aligning to the set of 791 reference genomes) increased with the number of samples used in the traditional co-assembly strategy (3.29 × 10^8^ ± 5.85 × 10^4^ base pairs [bp] for five-sample co-assemblies, 6.39 × 10^8^ ± 4.25 10^4^ bp for 12-sample co-assemblies, and 1.43 × 10^9^ ± 2.33 × 10^5^ bp for 48-sample co-assemblies) (mean ± SD) (Figure 1E and Table S1C). Despite the reduced assembly time and memory requirements, the mean aligned contig length for the sequential co-assemblies was 96.5% of that for the 48-sample co-assemblies (1.38 × 10^9^ ± 9.05 × 10^4^ bp, mean ± SD) (Figure 1E and Table S1C). The sequential co-assembly approach also recovered 99.7% of the 48-sample co-assembly mean recovered genome fraction (i.e., the percentage of bases in the 791 reference genomes with at least one co-assembled contig aligning to it) (46.06% ± 0.003% vs. 46.20% ± 0.004%, respectively, mean ± SD) (Figure 1F and Table S1C).

We observed that the number of MetaQUAST-classified misassembly events, which are identified by comparing the MEGAHIT assembled contigs to the 791 reference genomes, increased with the number of co-assembled reads (Figures 1G–1K). The sequential co-assemblies contained significantly fewer misassembly errors compared to the full 48-sample co-assemblies, as judged by the following metrics: (1) length of misassembled contigs (i.e., total number of bases in contigs co-assembled by MEGAHIT containing a misassembly; 1.02 × 10^8^ ± 1.66 × 10^6^ bp vs. 1.33 × 10^8^ ± 1.05 × 10^6^ bp, mean ± SD) (Figure 1G and Table S1C); (2) number of misassemblies (1.97 × 10^4^ ± 6.91 × 10^1^ vs. 2.35 × 10^4^ ± 1.12 × 10^2^, mean ± SD) (Figure 1H and Table S1C); (3) number of mismatches per 100 kilobase pairs (kbp) (i.e., average number of mismatches per 100,000 bases in the MEGAHIT assembled contigs aligning to the 791 reference genomes; 6.29 × 10^2^ ± 4.12 × 10^−1^ vs. 6.74 × 10^2^ ± 3.60 × 10^−1^, mean ± SD) (Figure 1I and Table S1C); and (4) number of local misassemblies (1.38 × 10^3^ ± 1.90 × 10^1^ vs. 1.95 × 10^3^ ± 1.68 × 10^1^, mean ± SD; see QUAST manual for more details) (Figure 1J and Table S1C) (*p* < 0.001 for all metrics, two-sample t test, Table S1D). The genome duplication ratios (total aligned bases from MEGAHIT assembled contigs divided by total aligned bases in the 791 reference genomes) were also lower for the sequential co-assemblies compared to the full 48-sample co-assemblies (1.035 vs. 1.074; Figure 1K and Table S1C). Notably, a trade-off of the sequential co-assembly approach is increased contig fragmentation. The traditional full 48-sample co-assembly yielded a greater mean total length for contigs exceeding 50 kbp than the sequential co-assembly (2.39 × 10^8^ ± 1.49 × 10^6^ bp vs. 1.62 × 10^8^ ± 1.56 × 10^6^ bp, mean ± SD) and a higher mean auN (a metric evaluating assembly contiguity, see QUAST manual^19^ for more details: 3.32 × 10^4^ ± 1.22 × 10^2^ vs. 2.22 × 10^4^ ± 6.63 × 10^1^ bp, mean ± SD) (Table S1E). Additional MetaQUAST metrics are listed in Table S1E.

Finally, we sought to evaluate co-assembly performance in the absence of reference genomes, given that the metagenomics datasets for which our strategy is designed will typically not have reference genomes. The distribution of sequencing reads representing each genome in a sample should approximate the relative abundances of the corresponding organisms in that sample. Therefore, we used the total percentage of reads aligning to an assembly as a metric of genome content recovery. Reads from all 48 samples were mapped to all of the co-assemblies (*n* = 3 replicates per each of the four co-assembly approaches) using Bowtie 2 (version 2.3.4.1, 12 CPUs per task) to obtain the overall read alignments (percentage of reads from each sample aligning to a given co-assembly: 78.37% ± 13.58% for the five-sample co-assemblies, 89.08% ± 9.62% for the 12-sample co-assemblies, 99.32% ± 0.39% for the sequential co-assemblies, and 99.48% ± 0.34% for the 48-sample traditional co-assemblies; mean ± SD) (Figure 1L and Table S1F). The overall read alignments for the sequential co-assemblies and the 48-sample co-assemblies were significantly higher than either the five-sample or 12-sample co-assemblies (*p* < 0.001, Kruskal-Wallis test with post hoc unpaired two-sample Wilcoxon tests; Table S1G). Although the 48-sample co-assemblies had a statistically significantly greater overall read alignment than the sequential co-assemblies (*p* < 0.001, unpaired two-sample Wilcoxon tests; Table S1G), the difference between the mean overall read alignments was only 0.16% (Table S1F).

We observed a linear relationship between the log-transformed mean recovered genome fraction and the mean overall read alignment for the four co-assembly strategies (linear model: mean overall read alignment [%] ∼ log(mean recovered genome fraction) [%], adjusted *R*^2^ = 0.996; Figure 1M and STAR Methods). This trend is likely the result of the cumulative relative abundances of the organisms within the community approximating a log distribution (Figure 1N and Table S1H). The dotplot in Figure 1N shows that the majority of reads within the 48 sequenced samples come from the most highly abundant organisms. Superimposed on this plot are four red dots representing values of the mean overall read alignment (Figure 1L and Table S1F) vs. the mean recovered genome fraction (Figure 1F and Table S1C) for each of the four co-assembly strategies. The cumulative relative abundance of the organisms within the community for a given genome fraction approximates the trend between the overall read alignment at a given recovered genome fraction. We also confirmed that sequential co-assembly does not significantly affect the recovery of low abundance genomes (e.g., *q* > 0.05, Dunn’s test) for the 79 genomes that represent the lowest relative abundances in the 791-genome dataset (Table S1I). Consequently, we proceeded to use overall read alignment as a proxy for evaluating genome recovery in samples without reference genome sets.

### Applying sequential co-assembly to shotgun sequencing reads from longitudinal human gut microbiome samples

We next evaluated our co-assembly strategy using sets of fecal-sample-derived Illumina NextSeq and NovaSeq shotgun sequencing reads from a clinical trial previously described by our lab.^20^ The trial was designed to investigate the effects of microbiota-directed complementary foods (MDCFs) in 12- to 18-month-old Bangladeshi children with moderate acute malnutrition. The study involved a 2-week pre-treatment phase in which participants consumed their typical diets followed by a 4-week feeding phase in which participants received an MDCF or a ready-to-use standard supplementary food (RUSF) twice daily for 4 weeks. The study concluded with a 2-week post-treatment phase in which participants returned to their original diets. Fecal samples were collected weekly throughout the study for a total of nine fecal samples per study participant. Analyses utilizing shotgun sequencing reads from these fecal samples are described in Gehrig et al.^20^

We selected reads from two sets of nine longitudinally collected fecal samples (4.80 × 10^7^ ± 2.56 × 10^7^ reads/sample for study participant 1219 and 4.57 × 10^7^ ± 2.19 × 10^7^ reads/sample for study participant 1339; mean ± SD) (Table S2A) to compare assembly of reads from these samples using our lab’s existing MAG assembly strategy, which involves co-assembling all reads from all nine collected samples per individual,^3^ to a parallel sequential co-assembly approach. Raw paired-end DNA-sequencing reads underwent quality control filtering and adapter trimming with TrimGalore^21^ (version 0.6.4) and host reads were removed with Bowtie 2 (version 2.3.4.1) (STAR Methods).

**Figure 2:**
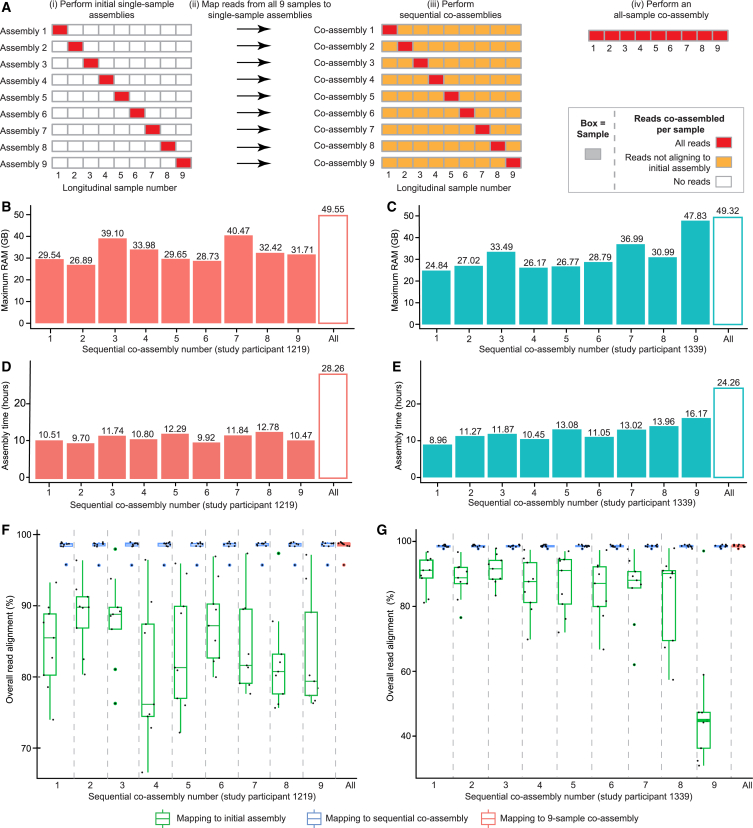
Comparing traditional and sequential co-assembly with longitudinal datasets from two study participants in a human gut microbiome study (A) Schematic outlining the strategies applied to assemble sequencing reads from each study participant’s nine samples. (B and C) Barplots of the maximum RAM (GB) utilized by MEGAHIT for each sequential co-assembly compared to the full nine-sample co-assembly for (B) study participant 1219 and (C) study participant 1339. (D and E) Barplots of the assembly time (hours) for each sequential co-assembly compared to the full nine-sample co-assembly for (D) study participant 1219 and (E) study participant 1339. (F and G) Boxplot of the overall read alignment (%) of reads mapping from all nine samples to each of the nine initial assemblies, the nine sequential co-assemblies, and the full nine-sample co-assembly for (F) individual 1219 and (G) individual 1339. Each dot in the boxplots represents the reads from one of the nine samples aligned to the assembly/co-assembly. Boxplots show minimum, first quartile, median, third quartile, and maximum values. See also Tables S2A–S2F.

Co-assemblies were generated from varying strategies for each study participant as shown in Figure 2A: (1) reads from each of the nine samples were assembled to yield nine single-sample assemblies; (2) reads from all nine samples were mapped to the nine single-sample assemblies; (3) nine sequential co-assemblies were generated by co-assembling reads from the initial sample plus reads from the nine samples not aligning to the single-sample assembly; and (4) reads from all nine samples were co-assembled using a “traditional” co-assembly approach. In total, this approach yielded 19 assemblies per study participant (nine single-sample assemblies, nine sequential co-assemblies, and one nine-sample co-assembly). All assemblies were generated with MEGAHIT (version 1.1.3; 8 CPUs per task). Reads were mapped using Bowtie 2 (version 2.3.4.1; 12 CPUs per task), and co-assemblies were evaluated using MetaQUAST (version 5.2.0) (STAR Methods).

Given that there were no reference genomes for these datasets, our evaluation focused on assembly time, computational resources, and overall read alignment. The maximum RAM utilized by MEGAHIT for a study participant’s sequential co-assemblies was less than that required for the nine-sample co-assembly (Figures 2B and 2C; Table S2B). Importantly, resource requirements for the set of nine sequential co-assemblies varied based on the initial assembled sample, with maximum RAM ranging from 26.89 to 40.47 GB for study participant 1219 and from 24.84 to 47.83 GB for study participant 1339 (Figures 2B and 2C; Table S2B).

Both the initial assembly step for the sequential co-assemblies and the assembly times for the sequential co-assemblies were faster than the co-assembly of all reads from all nine samples (1.97–4.80 h [single-sample assemblies] and 9.70–12.78 h [sequential co-assemblies] vs. 28.26 h [nine-sample co-assembly] for study participant 1219; 1.84–4.80 h [single-sample assemblies] and 8.96–16.17 h [sequential co-assemblies] vs. 24.26 h [nine-sample co-assembly] for study participant 1339) (Table S2B). Given that there were only nine samples per study participant, read mapping was performed in series, with the total amount of time to map all reads from all samples to each of the nine single-sample co-assemblies ranging from 7.77 to 9.85 h for study participant 1219 and from 6.46 to 8.89 h for study participant 1339 (Table S2B). Overall, the total computational time (initial assembly, read mapping, and sequential co-assembly) for 15 of the 18 sequential co-assemblies was less than the time required to assemble the nine-sample traditional co-assembly. Notably, the three sequential co-assemblies with longer overall computational times (all from participant 1339) only required an additional 6–99 min to complete (Table S2B). As observed with the simulated dataset, the “cost” of the sequential co-assembly was increased contig fragmentation compared to the traditional co-assembly; compared to the traditional nine-sample co-assembly, the 18 sequential co-assemblies across both study participants had lower lengths of contigs >50,000 bp, and 15 of the 18 sequential co-assemblies also had a lower auN (Table S2C).

Figures 2F and 2G show the distributions of overall read alignment for reads from the nine samples from each study participant aligning to (1) the series of initial one-sample assemblies (assemblies shown in Figure 2Ai), (2) the series of nine sequential co-assemblies (co-assemblies shown in Figures 2Aii and 2Aiii), and (3) the nine-sample co-assembly (co-assembly shown in Figure 2Aiv) (Table S2D). Comparing each single-sample co-assembly and the corresponding outputs from sequential co-assembly revealed that the overall read alignments for the sequential co-assembly were significantly higher (*p* < 0.001 for all comparisons, unpaired two-sample Wilcoxon test) (Figures 2F and 2G; Table S2E). Despite the wide range in overall read alignment for the initial assemblies, the overall read alignments for a study participant’s sequential co-assemblies were not significantly different from the full nine-sample co-assembly (*p* > 0.05 for each sequential co-assembly vs. the full nine-sample co-assembly, unpaired two-sample Wilcoxon test) (Figures 2F and 2G; Table S2F).

### Applying sequential co-assembly to a 1,600-sample, 2.3-terabyte dataset of shotgun sequencing microbiome data

Finally, we tested sequential co-assembly on a single computing node with a dataset consisting of 2.3 TB of shotgun sequencing data from our lab. This dataset was derived from a series of six gnotobiotic mouse experiments in which 8-week-old germ-free mice were colonized with a pooled microbial community created from 16 pre-treatment fecal samples obtained during the previously described clinical study^20^ (STAR Methods). A total of 94 diets were employed across six experiments, and 1,600 samples were collected in total, including (1) four aliquots (replicates) of the microbial communities from the pooled pre-intervention human fecal samples, (2) 263 cecal samples from gnotobiotic mice colonized with the community but exposed to different diets, (3) 1,325 gnotobiotic mouse fecal samples from the same experiments, and (4) eight adapter-only control samples. DNA was extracted from all samples and sequenced using either an Illumina NextSeq or NovaSeq instrument for an average depth of 1.07 × 10^7^ ± 2.80 × 10^6^ paired-end sequencing reads per sample (mean ± SD) (Table S3A and STAR Methods for description of read pre-processing and adapter trimming^21^), with 1.72 × 10^10^ paired-end sequencing reads in total across the 1,600 samples.

This dataset provided an opportunity to demonstrate how the selection of samples for initial co-assembly can affect the efficiency of the sequential co-assembly approach. Due to inherent differences in colonization efficiency, the relative abundances of members of a human gut microbiota will often change after transplantation into gnotobiotic animals.^22^ For example, *Prevotella* are frequently outcompeted by *Bacteroides* in the mouse gut.^22^ Because of this, we had reason to believe that the proportional representation of organisms in the human fecal sample-derived pooled microbial community would differ from those recovered in mouse cecal/fecal samples. Additionally, we postulated that the large number of diets consumed by the mice would result in a wide range of community compositions. Based on these considerations, we hypothesized that an initial co-assembly of reads derived from four samples of the pooled input microbial community may be ineffective for sequential co-assembly. We also reasoned that an initial co-assembly generated from reads of both the pooled input community samples and a small number of mouse samples could be effective, since the pooled input community may have greater representation of organisms that were present at lower abundance in the mouse-derived samples, and that the mouse samples may have greater representation of organisms at lower abundance than in the original pooled community. Additionally, we predicted that a sequential co-assembly would have a lower assembly time and fewer memory requirements than a full 1,600-sample co-assembly.

**Figure 3:**
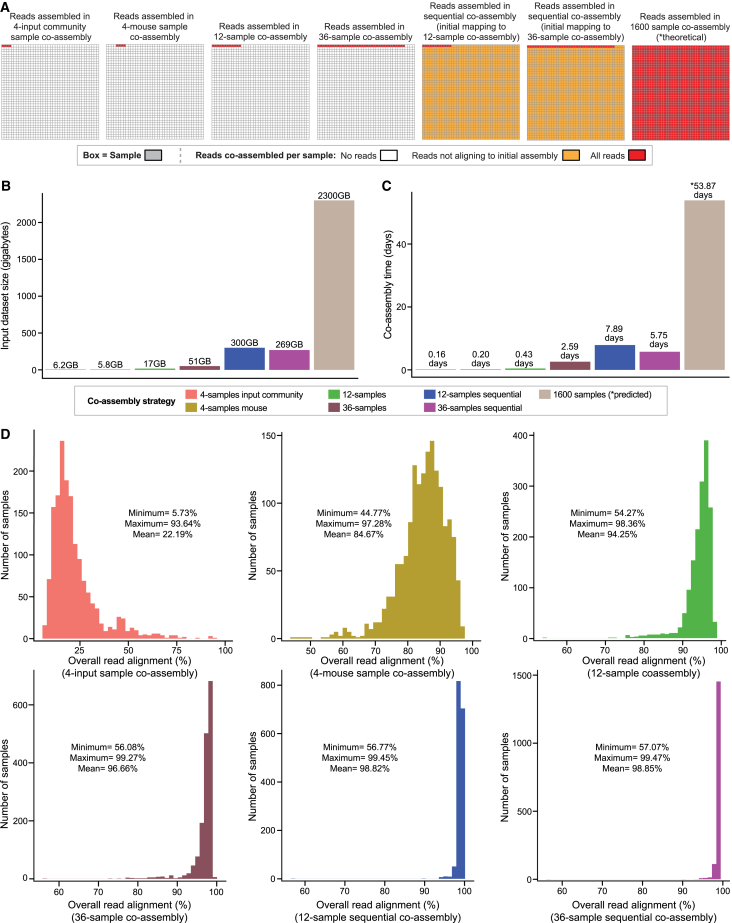
Applying sequential co-assembly to a 1,600-sample, 2.3-terabyte microbiome dataset from gnotobiotic mouse experiments (A) Schematic outlining the set of reads co-assembled for each of the co-assembly strategies. (B and C) Barplots showing (B) input dataset size (GB) and (C) co-assembly time (days) for each of the six co-assemblies. The time listed for the theoretical 1,600-sample co-assembly is a predicted value. (D) Histograms showing the total number of samples (out of 1,600) with a given overall read alignment (%) for each of the six co-assemblies. Minimum, maximum, and mean overall read alignment values (%) are shown on the inset of each histogram. See also Tables S3A–S3F.

We performed a series of six co-assemblies as shown in Figure 3A, including (1) a four-sample co-assembly that encompassed reads from the four replicates of the input pooled community derived from the human fecal samples, (2) a four-sample co-assembly that encompassed reads from four gnotobiotic mouse samples, (3) a 12-sample co-assembly that encompassed reads from the four replicates of the pooled community samples and eight gnotobiotic mouse samples, (4) a 36-sample co-assembly encompassing reads from the four replicates of the pooled community samples and 32 gnotobiotic mouse samples, (5) a sequential co-assembly that used the full set of reads from the 12 samples plus reads from all 1,600 samples not aligning to the initial 12-sample co-assembly, and (6) a sequential co-assembly that used the full set of reads from the 36 samples plus reads from all 1,600 samples that did not align to the initial 36-sample co-assembly. All assemblies were completed with MEGAHIT (version 1.1.3, 8 CPUs per task) on a single computing node. All mapping steps were completed with Bowtie 2 (version 2.3.4.1, 12 CPUs per task). Co-assemblies were evaluated based on metrics of overall input dataset size, co-assembly time, and overall read alignment (%). All contigs from the six co-assemblies were also evaluated using MetaQUAST (version 5.2.0) (STAR Methods).

The input dataset sizes were determined based on size of the gzipped, fastq R1 and R2 read files. The four-sample co-assemblies had the smallest input dataset size (6.2 and 5.8 GB) (Figure 3B and Table S3B). However, the datasets of reads not aligning to the four-sample pooled input community co-assembly and the four mouse sample co-assemblies were 500 GB and 1.9 TB, respectively; this made them too large for assembly by MEGAHIT and, therefore, sequential co-assembly was not feasible. In contrast, the 12-sample and 36-sample datasets were 17 GB and 51 GB, respectively; the datasets of reads not aligning to these two co-assemblies were 300 GB and 269 GB, respectively (i.e., only 13% and 11.7% the size of the full 1,600-sample dataset). These datasets were small enough to be assembled by MEGAHIT (Figure 3B and Table S3B), thereby enabling sequential co-assembly. The MetaQUAST metrics evaluating contig size and contiguity can be found in Table S3C.

Assembly time was shortest for each of the four-sample co-assemblies (0.16 and 0.20 days for the four-sample input pooled community samples and four-sample gnotobiotic mouse samples, respectively). Assembly time was 0.43 days for the 12-sample co-assembly and 2.59 days for the 36-sample co-assembly. The longest co-assembly times were for the two sequential co-assemblies (7.89 days and 5.75 days, respectively; Table S3B and Figure 3C). The assembly time for the various co-assembly strategies increased linearly with input dataset size (linear model of assembly time ∼ input data size, adjusted *R*^2^ = 0.94, STAR Methods). Based on this latter finding, we predicted that co-assembly of all reads from all 1,600 samples would be approximately 53.87 days (Figure 3C and Table S3B). Total computational times for the sequential co-assemblies (initial co-assembly, read mapping, and final co-assembly) were also significantly less than the predicted 1,600-sample co-assembly time. The maximum time required to map reads from one of the 1,600 samples to either the 12- or 36-sample co-assemblies was 45.27 min (Table S3D), whereas the cumulative read-mapping times for all 1,600 samples were 15.51 and 12.60 days, respectively. This yielded maximum total computational times of 23.83 days and 20.94 days (55.73% and 61.12% less than the predicted 53.87 days for the 1,600-sample co-assembly time).

Finally, the mean overall read alignments differed significantly between the four-pooled-input community and the four-mouse-sample co-assemblies (*p* < 0.001, unpaired two-sample Wilcoxon test; 22.19% ± 12.52% [mean ± SD] overall read alignment for reads aligning to the four-pooled-input community sample co-assembly; 84.67% ± 7.25% overall read alignment for reads aligning to the four-mouse-sample co-assembly) (Figure 3D; Tables S3E and S3F). The 36-sample sequential co-assembly achieved the greatest overall read alignment among the six strategies across the 1,600 samples (*p* < 0.001 for the sequential co-assembly vs. the four-sample, 12-sample, 36-sample, and 12-sample sequential co-assemblies; unpaired two-samples Wilcoxon tests; 98.85% ± 1.22% [mean ± SD]) (Figure 3D; Tables S3E and S3F). Overall, these results highlight the magnitude of assembly time reduction that sequential co-assembly can achieve with terabyte-scale datasets.

## Discussion

The strategy described here addresses a specific challenge in metagenomics: how to efficiently deploy computational resources to co-assemble reads from large, multi-sample datasets. Overall, we have demonstrated that the removal of redundant sequencing reads through a sequential co-assembly approach can significantly reduce dataset size, thereby reducing memory requirements, time required for assembly, and overall computational time for both simulated and actual biological datasets. Notably, this approach can also be used to assemble terabyte-scale metagenomic datasets on a single-node computing system. Available metrics of assembly accuracy with simulated datasets suggest that the sequential method reduces assembly errors and yields similar levels of rare genome recovery compared to the traditional assembly method. One trade-off of the sequential methodology is increased fragmentation, as was seen in the majority of cases presented in this study.

A priori knowledge of the compositional similarity of microbial community samples being assessed can be used to optimize the performance of sequential co-assembly. This is because the greater the overall read alignment of samples to the initial co-assembly, the greater the reduction in duplicate reads and assembly time. As shown with our experiments in gnotobiotic mice, strategically choosing samples that contain varying degrees of coverage of low- and high-abundance organisms can help achieve a more complete initial assembly. Although it is not always possible to know which samples may or may not be effective for the initial assembly step, using longitudinally sampled human microbiota datasets, we show that regardless of the initial assembled sample, the overall read alignments for the series of sequential co-assemblies were nearly identical. This suggests that if a key goal of analysis is to generally reduce overall assembly time and memory requirements, choosing any set of representative samples for the initial assembly may be feasible. If the goal is to have the greatest possible reduction in assembly time and memory requirements, further optimization could be achieved by performing a series of multiple initial assemblies and proceeding with the assembly yielding the greatest overall read alignment for the sequential co-assembly process, as demonstrated with our 1,600-sample dataset.

Although MEGAHIT was used as the main assembler for this series of analyses, in principle, sequential co-assembly can be integrated into any assembly workflow. It is worth noting that since this approach utilizes an assembler and read-mapping program for deduplication efforts, it reduces the total number of unique computational programs required during this portion of a metagenomics pipeline. Additionally, while co-assembly is not desirable for every metagenomics dataset, sequential co-assembly may increase accessibility for co-assembly of datasets that were previously too large or would be too time intensive to be handled with single-node computing structures.^16^ Increasing accessibility of large-scale sample co-assemblies for the metagenomics community is especially critical when considering that this strategy would improve the feasibility of sample co-assembly in computationally limited resource settings, such as in low- and middle-income countries, where there is a push for greater development and implementation of bioinformatics platforms for microbiome studies.^13^

### Limitations of the study

The time and computational efficiencies afforded by the sequential co-assembly approach are dependent on the representation of duplicate reads within a dataset. Datasets with high degrees of duplication have the potential for greater reductions in overall assembly time and memory requirements compared to datasets with lower duplication rates. However, the reduction in assembly errors compared to traditional co-assembly suggests benefits for datasets over a broad range of sizes. Additionally, the metric of overall read alignment works under the assumption that the community has been sequenced deeply enough to capture all organisms. If all samples are only sequenced deeply enough to capture the most highly abundant organisms, and no reads are captured from the low-abundance organisms, the overall read alignment may appear to be artificially high and miss potential alignment to low-abundance organisms. Moreover, there is a risk of reads from repetitive or shared regions across genomes being artificially removed if regions in the genomes from the first co-assembly contain identical sequences. This approach also does not evenly reduce duplicate reads across organisms. It is biased toward removing redundant reads from more highly abundant organisms in the community. Further optimization of the strategy will be needed to achieve more even deduplication across organisms. Running an existing deduplication method, such as fastp or bbtools dedup, on reads not aligning to the initial co-assembly may help to achieve even greater reductions in duplicate reads and assembly time.

## Resource availability

### Lead contact

Requests for further information and resources should be directed to and will be fulfilled by the lead contact, Jeffrey I. Gordon (jgordon@wustl.edu).

### Materials availability

No new reagents were generated in this study.

### Data and code availability

• Simulated shotgun sequencing data from the CAMI mouse gut dataset were published previously. The data are available at PUBLISSO: https://doi.org/10.4126/FRL01-006421672. Quality-controlled, adapter-trimmed, host-filtered, paired-end shotgun sequencing reads derived from 18 human fecal samples and 1,600 samples from gnotobiotic mice are available at the Sequence Read Archive (SRA): BioProject PRJNA1137057.
• Code for all computational tasks and analyses is available on Zenodo: https://doi.org/10.5281/zenodo.14796098.
• Any additional information required to reanalyze data reported in this paper is available from the lead contact upon request.

## Acknowledgments

We thank Matthew Hibberd, Daniel Webber, Nathaniel McNulty, and Michael Barratt for providing valuable insights and feedback in the development of the manuscript; Daniel Webber, David O’Donnell, Maria Karlsson, Justin Serugo, and Hao-Wei Chang for assisting in conducting the gnotobiotic mouse experiments used to generate biospecimens for the gnotobiotic mouse experiment sequencing dataset; and Steven Hartman for helping with pre-processing longitudinal human shotgun sequencing reads. We are indebted to Eric Martin and Brian Koebbe for providing technical support with the Washington University Center for Genome Sciences and Systems Biology High Throughput Computing Facility computational cluster; Martin Meier for performing DNA extractions; Jessica Hoisington-Lopez and MariaLynn Crosby in the DNA Sequencing Innovation Lab at Washington University School of Medicine; and members of the Genome Technology Access Center at Washington University School of Medicine for performing the shotgun sequencing. This work was supported by the 10.13039/100000865Bill and Melinda Gates Foundation (OPP1196579), the NIH (DK30292), the Medical Scientist Training Program NIH grant (5T32GM007200) at Washington University in St. Louis, and the Washington University in St. Louis James Miller and Cora Swift Miller graduate student scholarship. H.M.L. is a member of the Medical Scientist Training Program at Washington University School of Medicine and recipient of the James Miller and Cora Swift Miller graduate student scholarship. The graphical abstract was created using BioRender (Lynn, H. [2025] https://BioRender.com/h47r773). Final modifications to the graphical abstract were made using Adobe Illustrator.

## Author contributions

H.M.L. conceptualized the sequential co-assembly idea, designed and performed gnotobiotic mouse experiments used to generate the 1,600-sample sequencing dataset, and performed all computational tasks described in this paper. J.I.G. oversaw the studies described. H.M.L. and J.I.G. wrote the manuscript.

## Declaration of interests

The authors declare no competing interests.

## STAR★Methods

### Key resources table

**Table:** 

REAGENT or RESOURCE	SOURCE	IDENTIFIER
**Deposited data**		

HiSeq simulated mouse gut dataset	Meyer et al.^17^	PUBLISSO: https://repository.publisso.de/resource/frl:6421672
Shotgun sequencing reads from 18 human fecal samples	This paper	Sequence Read Archive: Bio Project PRJNA1137057
Shotgun sequencing reads from 1,600 gnotobiotic mouse experiment samples	This paper	Sequence Read Archive: Bio Project PRJNA1137057
UCSC mm10 mouse genome	NCBI assembly database	https://hgdownload.soe.ucsc.edu/downloads.html
UCSC hg19 human genome	NCBI assembly database	https://hgdownload.soe.ucsc.edu/downloads.html

**Software and algorithms**		

samtools	Danecek et al.^9^	https://www.htslib.org/doc/samtools-fasta.html
MEGAHIT	Li et al.^15^	https://github.com/voutcn/megahit
Bowtie 2	Langmead and Salzberg^18^	http://bowtie-bio.sourceforge.net/bowtie2/index.shtml
MetaQUAST	Mikheenko et al.^19^	https://quast.sourceforge.net/metaquast
Trim Galore	Krueger et al.^21^	https://github.com/FelixKrueger/TrimGalore
Cutadapt	Martin^23^	https://cutadapt.readthedocs.io/en/stable/
RStudio	RStudio team^24^	http://www.rstudio.com/
R	R core team^25^	https://www.R-project.org
ggplot2	Wickham^26^	https://ggplot2.tidyverse.org/
tidyverse	Wickham et al.^27^	https://www.tidyverse.org/
dplyr	Wickham et al.^28^	https://dplyr.tidyverse.org/
FSA: Simple Fisheries Stock Assessment Methods	Ogle et al.^29^	https://cran.r-project.org/web/packages/FSA/index.html
Code used for all computational tasks and analyses shown in this paper	This paper	https://doi.org/10.5281/zenodo.14796098

### Experimental model and study participant details

#### Simulated HiSeq sequencing data

The description of the toy mouse gut microbiome dataset is found on the main CAMI website: https://data.cami-challenge.org/participate.

#### Human microbiome clinical trial

The shotgun sequencing data was derived from a clinical trial previously described by our lab and the full set of details of the clinical trial (ClinicalTrials.gov identifier *NCT03084731*) including the primary and secondary outcomes, ethics statements, informed consent, reporting of harm, sample size determination, funding sources, and conflicts of interest have been previously published.^20^ Briefly, this study was designed to test the effects of three lead microbiota directed complementary foods (MDCFs) compared to a ready to use supplementary food (RUSF) in 12-18-month-old Bangladeshi children. 63 children were enrolled across the four treatment arms and the study protocol involved a two-week pre-intervention phase, a four-week intervention phase in which participants were randomly assigned to receive two servings per day of one of the four supplemental foods, and a two-week post-intervention follow-up. Fecal samples were collected weekly throughout the study for a total of nine samples collected per study participant.

#### Gnotobiotic mouse experiments

All gnotobiotic mouse experiments utilized germ-free C57BL/6J mice and were performed in accordance with protocols approved by the Washington University Animal Studies Committee. A total of 263 8-week-old mice were used across the six experiments and mice were colonized with a pooled microbial community derived from 16 fecal samples collected prior to intervention in the aforementioned clinical trial. Three of the six experiments utilized only male mice, for a total of 98 males. The remaining three experiments utilized 165 animals in total, with 83 males and 82 females.

### Method details

#### Simulated shotgun sequencing dataset

##### Data download

Tar files containing simulated interleaved HiSeq reads for 48 simulated mouse gut samples were downloaded from https://frl.publisso.de/data/frl:6421672/dataset/2017.12.29_11.37.26_sample_[number]_reads.tar. The read files were opened using the command “tar -xvf”.

##### Assembly of reads

The sequencing reads from the following samples were used for each of the co-assemblies in Figure 1:

(1) 5-sample co-assembly: samples 0, 1, 2, 5, and 6.
(2) 12-sample co-assembly: samples 0, 1, 2, 5, 6, 7, 8, 9, 10, 11, 12, and 14.
(3) 48-sample co-assembly: samples 0, 1, 2, 5, 6, 7, 8, 9, 10, 11, 12, 14, 15, 16, 17, 18, 19, 20, 21, 22, 23, 24, 25, 26, 27, 28, 29, 30, 31, 32, 33, 34, 35, 36, 37, 38, 52, 53, 54, 55, 56, 57, 58, 59, 60, 61, 62, 63.
(4) Sequential co-assembly: samples 0, 1, 2, 5, and 6, plus reads from all 48 samples not aligning to the 5-sample co-assembly.

MEGAHIT^15^ (version 1.1.3: 8 CPUs per task, --mem-flag 1) was used for both the initial co-assemblies and the sequential co-assemblies. Each co-assembly strategy was run in triplicate to generate 12 co-assemblies in total.

The sequential co-assembly involved additional steps. First, a mapping index was generated for the five-sample co-assemblies using Bowtie 2^18^ command “Bowtie 2-build” (version 2.3.4.1, 12 CPUs per task). Reads from all 48 samples were mapped to the Bowtie index using the “Bowtie 2” command. Unconcordant reads were identified with the inclusion of the flag “--un-conc-gz”. Samtools^9^ (version 1.9), was used to generate corresponding bam files for the unconcordant reads (samtools view -hbSF4). For each sequential co-assembly, the unconcordantly aligning reads were concatenated together, along with the reads from the original five samples, and assembled using MEGAHIT as described above. The values reported in Figure 1 and Table S1 were obtained using the following commands:

• Figure 1B and Table S1A, MaxRSS: sacct -j [ID].batch -o jobid, reqmem,maxrss --units = G.
• Figure 1C and Table S1A, total memory for reads: cat [MEGAHIT log file] | grep “Memory for reads”.
• Figure 1D and Table S1A, assembly time: cat [MEGAHIT log file] | grep “ALL DONE. Time elapsed”.
• Table S1B, Bowtie 2 mapping times: “sacct -j [Bowtie 2 job ID] --format = Elapsed”.

##### MetaQUAST evaluation of co-assemblies

MetaQUAST^19^ was used to compare the twelve sets of contigs to the set of 791 reference genomes from which the simulated HiSeq data had been derived. The MetaQUAST version 5.2.0 package was downloaded from https://github.com/ablab/quast/releases/download/quast_5.2.0/quast-5.2.0.tar.gz and the set of 791 reference genomes corresponding with the assembled reads were downloaded from https://frl.publisso.de/data/frl:6421672/dataset/CAMISIM_setup.tar.gz. The CAMISIM_setup.tar.gz file contained a “source genomes” folder containing the fasta copies of the reference genomes. MetaQUAST was run within the downloaded 5.2.0 folder (“./metaquast.py” command) with the following parameters as described in the CAMI tutorial^17^: --reuse-combined-alignments, --no-icarus, -r 791_genomes, -t 28, --unique-mapping. Metrics comparing the contig assemblies to the set of reference genomes, as well as metrics independent of the reference genomes, in Figures 1E–1K and Tables S1C and S1E were obtained from the MetaQUAST “report.tsv” files.

##### Cumulative relative abundance calculations

Relative abundances of the 791 genomes across the 48 samples were calculated from absolute abundance values reported in the “CAMISIM_setup” folder and corresponding “distribution.txt” files.

##### Evaluation of assemblies with read alignment

A mapping index was generated for each of the twelve co-assemblies using Bowtie 2 command “Bowtie 2-build” (version 2.3.4.1 and 12 CPUs per tasks). Reads from all 48 samples were mapped to each of the 12 co-assembly indices using Bowtie 2 (version 2.3.4.1) command “Bowtie 2”. Unconcordant reads were identified with the flag “--un-conc-gz”. Samtools (version 1.9) was used to generate corresponding bam files for the unconcordant reads (samtools view -hbSF4). The “overall read alignment” values shown in the histograms in Figure 1L were obtained from the corresponding Samtools summaries using the following command: cat [slurm_file_name].out | grep “overall alignment rate”. We obtained the recovered genome fraction for each of the 791 genomes (Table S1H) from the MetaQUAST “summary/TXT” folder, “Genome_fraction.txt” document.

#### Human fecal sample sequencing dataset

##### Data download

Quality controlled, host-filtered shotgun sequencing data from the 18 fecal samples used in this analysis can be found at the Sequence Read Archive (BioProject number PRJNA1137057). The nine samples used from study participant 1219 include: 1219101G_HF_CAT_R1/2.fq.gz, 1219102G_HF_CAT_R1/2.fq.gz, 1219103G_HF_CAT_R1/2.fq.gz, 1219104G_HF_CAT_R1/2.fq.gz, 1219105G_HF_CAT_R1/2.fq.gz, 1219106G_HF_CAT_R1/2.fq.gz, 1219107G_HF_CAT R1/2.fq.gz, 1219108G_HF_CAT_R1/2.fq.gz, 1219109G_HF_CAT R1/2.fq.gz. The nine samples used from study participant 1339 include: 1339101G_HF_CAT_R1/2.fq.gz, 1339102G_HF_CAT_R1/2.fq.gz, 1339103G_HF_CAT R1/2.fq.gz, 1339104G_HF_CAT R1/2.fq.gz, 1339105G_HF_CAT_R1/2.fq.gz, 1339106G_HF_CAT R1/2.fq.gz, 1339107G_HF_CAT_R1/2.fq.gz, 1339108G_HF_CAT _R1/2.fq.gz, 1339109G_HF_CAT_R1/2.fq.gz.

Briefly, DNA extraction and library preparation were performed as previously described^20^ and reads were sequenced using Illumina NovaSeq 6000 or NextSeq 500. Raw reads underwent quality filtering to remove reads <50bp, and adapter trimming using TrimGalore^21^ (version: 0.6.4, 4 cores for trimming, ASCII+33 quality encoding, ‘CTGTCTCTTATA’ adapter sequence, --nextseq-trim = 20) with Cutadapt^23^ (version: 1.16) and Python version 3.5.2. Host reads were removed from the quality controlled and trimmed reads by only taking reads not aligning to the host genome (Bowtie 2 version 2.3.4.1; UCSC hg19 human genome, --un-conc-gz).

##### Assembly of reads

MEGAHIT (version 1.1.3, 8 CPUs per task, --mem-flag 1) was used for all co-assembly steps including the initial single-sample assemblies, all sequential co-assemblies, and the full 9-sample co-assembly. Sequential co-assembly involved additional steps of generation of a mapping index from the single sample co-assemblies, mapping reads from all nine samples to the initial single-sample co-assemblies with Bowtie 2 and a subsequent co-assembly of initial sample reads plus reads not aligning to the initial co-assemblies from all nine samples with MEGAHIT using the commands described in the previous section. The values reported in Figure 2 and Table S2B were obtained using the following commands:

• Figures 2B, 2C and Table S2B - RAM: sacct -j [ID].batch -o jobid, reqmem,maxrss --units = G”.
• Figures 2D, 2E and Table S2B - assembly time: cat [MEGAHITlog file] | grep “ALL DONE. Time elapsed”.
• Table S2B- read mapping time: “sacct -j [slurm jobid] --format = Elapsed”.

##### MetaQUAST evaluation of co-assemblies

MetaQUAST was run within the downloaded 5.2.0 folder (“./metaquast.py” command) using the following parameters: --reuse-combined-alignments, --no-icarus, -t 28, --unique-mapping. Metrics comparing the contig assemblies in Table S2C were obtained from the MetaQUAST “report.tsv” files.

##### Read-alignment evaluation of co-assemblies

A mapping index was generated from the nine original single-sample assemblies, nine sequential co-assemblies, and full nine-sample co-assembly for each study participant using Bowtie 2 command “Bowtie 2-build” (version 2.3.4.1 and 12 CPUs per tasks). Reads from all nine samples per individual were mapped to that individual’s Bowtie indices using Bowtie 2 (version 2.3.4.1) command “Bowtie 2”. Unconcordant reads were identified with the flag --un-conc-gz. Samtools (version 1.9) was used to generate corresponding bam files for the unconcordant reads (samtools view -hbSF4).

The “overall read alignment” values shown in the histograms in Figures 2F and 2G and listed in Table S2D were obtained from the corresponding Samtools summaries using the following command: cat [slurm_file_name].out | grep “overall alignment rate”.

#### Mouse experiments sequencing dataset

##### Data overview

A total of 1,600 samples from the gnotobiotic mouse experiments were sequenced and include the following sample types: (i) 4 aliquots of the microbial communities in the pooled pre-intervention human fecal samples used to colonize the gnotobiotic mice; (ii) 263 gnotobiotic mouse cecal samples; (iii) 1325 serially collected gnotobiotic mouse fecal samples; and (iv) 8-adapter only samples.

##### DNA shotgun sequencing

Quality controlled, host-filtered shotgun sequencing reads from the 1,600 samples can be found on the Sequence Read Archive (BioProject number PRJNA1137057). Briefly, raw DNA sequencing reads from all 1,600 samples were generated with Illumina NextSeq 500 or NovaSeq 6000 instruments. Reads were demultiplexed with bcl2fastq (Illumina) and underwent pre-processing for quality control and adapter trimming with TrimGalore (version 0.6.4) using Cutadapt (version 1.16) and Python (version 3.5.2). Host reads were removed from the quality controlled and trimmed reads by taking only reads not mapping to the host genome with Bowtie 2 version 2.3.4.1 (UCSC hg19 human genome for the pooled microbial communities from human fecal samples and UCSC mm10 for all mouse fecal and cecal samples, --un-conc-gz to isolate reads not aligning to the host genomes).

##### Assembly of reads

MEGAHIT (version 1.1.4, 8 CPUs per task, --mem-flag 1) was used for all co-assembly steps including both 4-sample co-assemblies, the 12-sample and 36-sample co-assemblies, and both sequential co-assemblies. The following paired-end shotgun sequencing read files were used for each of the co-assembly strategies:

• Four pooled community sample co-assembly: Sample_1_HF_CAT_R1/R2.fq.gz, Sample_2_HF_CAT_R1/R2.fq.gz, Sample_370_HF_CAT_R1/R2.fq.gz, Sample_371_HF_CAT_R1/R2.fq.gz.
• Four mouse gnotobiotic mouse sample co-assembly: Sample_437_HF_CAT_R1/R2.fq.gz, Sample_453_HF_CAT_R1/R2.fq.gz, Sample_470_HF_CAT_R1/R2.fq.gz, Sample_486_HF_CAT_R1/R2.fq.gz.
• 12-sample co-assembly: Sample_1_HF_CAT_R1/R2.fq.gz, Sample_2_HF_CAT_R1/R2.fq.gz, Sample_160_HF_CAT_R1/R2.fq.gz, Sample_233_HF_CAT_R1/R2.fq.gz, Sample_260_HF_CAT_R1/R2.fq.gz, Sample_264_HF_CAT_R1/R2.fq.gz,
• Sample_370_HF_CAT_R1/R2.fq.gz, Sample_371_HF_CAT_R1/R2.fq.gz, Sample_437_HF_CAT_R1/R2.fq.gz, Sample_453_HF_CAT_R1/R2.fq.gz, Sample_470_HF_CAT_R1/R2.fq.gz, Sample_486_HF_CAT_R1/R2.fq.gz.
• 36-sample co-assembly: Sample_1_HF_CAT_R1/R2.fq.gz, Sample_2_HF_CAT_R1/R2.fq.gz, Sample_107_HF_CAT_R1/R2.fq.gz, Sample_160_HF_CAT_R1/R2.fq.gz, Sample_162_HF_CAT_R1/R2.fq.gz, Sample_178_HF_CAT_R1/R2.fq.gz, Sample_233_HF_CAT_R1/R2.fq.gz, Sample_249_HF_CAT_R1/R2.fq.gz, Sample_260_HF_CAT_R1/R2.fq.gz, Sample_264_HF_CAT_R1/R2.fq.gz, Sample_348_HF_CAT_R1/R2.fq.gz, Sample_370_HF_CAT_R1/R2.fq.gz, Sample_371_HF_CAT_R1/R2.fq.gz, Sample_437_HF_CAT_R1/R2.fq.gz, Sample_453_HF_CAT_R1/R2.fq.gz, Sample_470_HF_CAT_R1/R2.fq.gz, Sample_483_HF_CAT_R1/R2.fq.gz, Sample_486_HF_CAT_R1/R2.fq.gz, Sample_526_HF_CAT_R1/R2.fq.gz, Sample_537_HF_CAT_R1/R2.fq.gz, Sample_630_HF_CAT_R1/R2.fq.gz, Sample_636_HF_CAT_R1/R2.fq.gz, Sample_664_HF_CAT_R1/R2.fq.gz, Sample_706_HF_CAT_R1/R2.fq.gz, Sample_773_HF_CAT_R1/R2.fq.gz, Sample_796_HF_CAT_R1/R2.fq.gz, Sample_906_HF_CAT_R1/R2.fq.gz, Sample_1009_HF_CAT_R1/R2.fq.gz, Sample_1038_HF_CAT_R1/R2.fq.gz, Sample_1061_HF_CAT_R1/R2.fq.gz, Sample_1121_HF_CAT_R1/R2.fq.gz, Sample_1122_HF_CAT_R1/R2.fq.gz, Sample_1162_HF_CAT_R1/R2.fq.gz, Sample_1393_HF_CAT_R1/R2.fq.gz, Sample_1568_HF_CAT_R1/R2.fq.gz, Sample_1571_HF_CAT_R1/R2.fq.gz.
• Sequential 12-sample co-assembly: Sample_1_HF_CAT_R1/R2.fq.gz, Sample_2_HF_CAT_R1/R2.fq.gz, Sample_160_HF_CAT_R1/R2.fq.gz, Sample_233_HF_CAT_R1/R2.fq.gz, Sample_260_HF_CAT_R1/R2.fq.gz, Sample_264_HF_CAT_R1/R2.fq.gz,
• Sample_370_HF_CAT_R1/R2.fq.gz, Sample_371_HF_CAT_R1/R2.fq.gz, Sample_437_HF_CAT_R1/R2.fq.gz, Sample_453_HF_CAT_R1/R2.fq.gz, Sample_470_HF_CAT_R1/R2.fq.gz, Sample_486_HF_CAT_R1/R2.fq.gz, plus reads from all 1,600 samples not aligning to the 12-sample co-assembly.
• Sequential 36-sample co-assembly: Sample_1_HF_CAT_R1/R2.fq.gz, Sample_2_HF_CAT_R1/R2.fq.gz, Sample_107_HF_CAT_R1/R2.fq.gz, Sample_160_HF_CAT_R1/R2.fq.gz, Sample_162_HF_CAT_R1/R2.fq.gz, Sample_178_HF_CAT_R1/R2.fq.gz, Sample_233_HF_CAT_R1/R2.fq.gz, Sample_249_HF_CAT_R1/R2.fq.gz, Sample_260_HF_CAT_R1/R2.fq.gz, Sample_264_HF_CAT_R1/R2.fq.gz, Sample_348_HF_CAT_R1/R2.fq.gz,
• Sample_370_HF_CAT_R1/R2.fq.gz, Sample_371_HF_CAT_R1/R2.fq.gz, Sample_437_HF_CAT_R1/R2.fq.gz, Sample_453_HF_CAT_R1/R2.fq.gz, Sample_470_HF_CAT_R1/R2.fq.gz, Sample_483_HF_CAT_R1/R2.fq.gz, Sample_486_HF_CAT_R1/R2.fq.gz, Sample_526_HF_CAT_R1/R2.fq.gz, Sample_537_HF_CAT_R1/R2.fq.gz, Sample_630_HF_CAT_R1/R2.fq.gz, Sample_636_HF_CAT_R1/R2.fq.gz, Sample_664_HF_CAT_R1/R2.fq.gz, Sample_706_HF_CAT_R1/R2.fq.gz, Sample_773_HF_CAT_R1/R2.fq.gz, Sample_796_HF_CAT_R1/R2.fq.gz, Sample_906_HF_CAT_R1/R2.fq.gz, Sample_1009_HF_CAT_R1/R2.fq.gz, Sample_1038_HF_CAT_R1/R2.fq.gz, Sample_1061_HF_CAT_R1/R2.fq.gz, Sample_1121_HF_CAT_R1/R2.fq.gz, Sample_1122_HF_CAT_R1/R2.fq.gz, Sample_1162_HF_CAT_R1/R2.fq.gz, Sample_1393_HF_CAT_R1/R2.fq.gz, Sample_1568_HF_CAT_R1/R2.fq.gz, Sample_1571_HF_CAT_R1/R2.fq.gz, plus reads from all 1,600 samples not aligning to the 36-sample co-assembly.

Sequential co-assembly involved additional steps of read mapping with Bowtie 2 and a subsequent co-assembly with MEGAHIT as described above. The values reported in Figure 3 and listed in Tables S3B and S3C were obtained using the following commands:

• Figure 3B and Table S3B- Input data size: “ll” (within the reads file folder).
• Figure 3C and Table S3B - Assembly time: cat [MEGAHIT log file] | grep “ALL DONE. Time elapsed”.
• Table S3D - Bowtie 2 mapping time: “sacct -j [slurm jobid] --format = Elapsed”.

##### MetaQUAST evaluation of co-assemblies

MetaQUAST was run within the downloaded 5.2.0 folder (“./metaquast.py” command) with the following parameters: --reuse-combined-alignments, --no-icarus, -t 28, --unique-mapping. Metrics comparing the contig assemblies in Table S3C were obtained from MetaQUAST “report.tsv” files.

##### Read alignment evaluation of assemblies

A mapping index was generated for each of the co-assemblies using Bowtie 2 command “Bowtie 2-build” (version 2.3.4.1 and 12 CPUs per tasks). Reads from all 1,600 samples per individual were mapped to the four co-assembly Bowtie indices using Bowtie 2 (version 2.3.4.1) command “Bowtie 2”. Unconcordant reads were identified with the flag --un-conc-gz. Samtools (version 1.9) was used to generate corresponding bam files for the unconcordant reads (samtools view -hbSF4). The “overall read alignment” values shown in the histograms in Figure 3D and Table S3E were obtained from the corresponding samtools summaries using the following command: cat [slurm_file_name].out | grep “overall alignment rate”.

### Quantification and statistical analysis

All statistical tests (results reported in Tables S1D, S1G, S1I, S2E, S2F, and S3F) were performed in RStudio^24^ (version 2022.7.2, build 576), R^25^ version 4.2.2. Plots in Figures 1, 2, and 3 were generated in RStudio (version 2022.7.2, build 576), R version 4.2.2, with the ggplot2^26^ package (version 3.4.0). Packages used for data handling included tidyverse^27^ (version 1.3.2) and dplyr^28^ (version 1.1.3). Statistical significance was defined as *p* < 0.05. Data were evaluated for normality using the command “shapiro.test” and equal variance using the command “var.test”. After normality (*p* > 0.05) and equal variance (*p* > 0.05) were confirmed, two-sample t-test results in Table S1D were calculated with *n* = 3 co-assemblies per assembly strategy using the command “t.test” with the flag “var.equal = TRUE”. The Kruskal-Wallis and unpaired two-sample Wilcoxon test in Table S1G were performed on *n* = 48 samples’ reads mapped to a given co-assembly using the commands “kruskal.test” and “wilcox.test”. The Kruskal-Wallis and post-hoc Dunn test in Table S1I were performed on *n* = 79 genomes per co-assembly strategy using the commands: “kruskal.test” and “dunnTest”.^29^ The unpaired two-sample Wilcoxon tests performed in Tables S2E and S2F were performed on *n* = 9 overall read alignments (one for each of the 9 samples) per co-assembly strategy using the command “wilcox.test”. The unpaired two-sample Wilcoxon tests in Table S3F were performed on *n* = 1,600 overall read alignments (one for each of the 1,600 samples) per co-assembly strategy using the command “wilcox.test”. All reports of mean and standard deviation were determined in R using the commands “mean” and “stdev”. We fit a linear model (R “lm” command) to determine the relationship between mean overall read alignment and log (mean recovered genome fraction) for the four co-assemblies from the 48-sample simulated dataset, as well as for co-assembly time and dataset size for the 6 co-assemblies from the 1,600 sample gnotobiotic mouse experiments dataset. Adjusted R^2^ values were extracted from the model using the “summary” command.

## Supplemental information

Document S1. Figure S1

Table S1. Tables for co-assembly of simulated Illumina shotgun sequencing reads from 48 representative mouse gut microbiome samples, related to Figure 1

Table S2. Tables for co-assembly of shotgun sequencing reads from longitudinal human gut microbiome samples, related to Figure 2

Table S3. Tables for co-assembly of 1,600 samples from gnotobiotic mouse studies, related to Figure 3

Document S2. Article plus supplemental information
