# Supplementary material for: Sequential co-assembly reduces computational resources and errors in metagenome-assembled genomes
Source: Cell Rep Methods. 2025 Mar 17;5(3):101005. doi: 10.1016/j.crmeth.2025.101005 (PMC12049710; doi:10.1016/j.crmeth.2025.101005)
Supplement: Document S1. Figure S1 [file mmc1.pdf]

Cell Reports Methods, Volume *5*

Supplemental information

Sequential co-assembly reduces computational resources and errors in metagenome-assembled genomes

Hannah M. Lynn and Jeffrey I. Gordon

A

Bacteria relative abundance	Sample 1	Sample 2	Sample 3	Sample 4	Sample 5	Sample 6	Sample 7	Sample 8	Sample 9	Sample 10
○	90%	90%	90%	90%	90%	90%	90%	90%	90%	90%
○	10%	0%	10%	0%	10%	0%	0%	0%	10%	10%
○	0%	0%	0%	10%	0%	0%	10%	0%	0%	0%
○	0%	10%	0%	0%	0%	10%	0%	10%	0%	0%

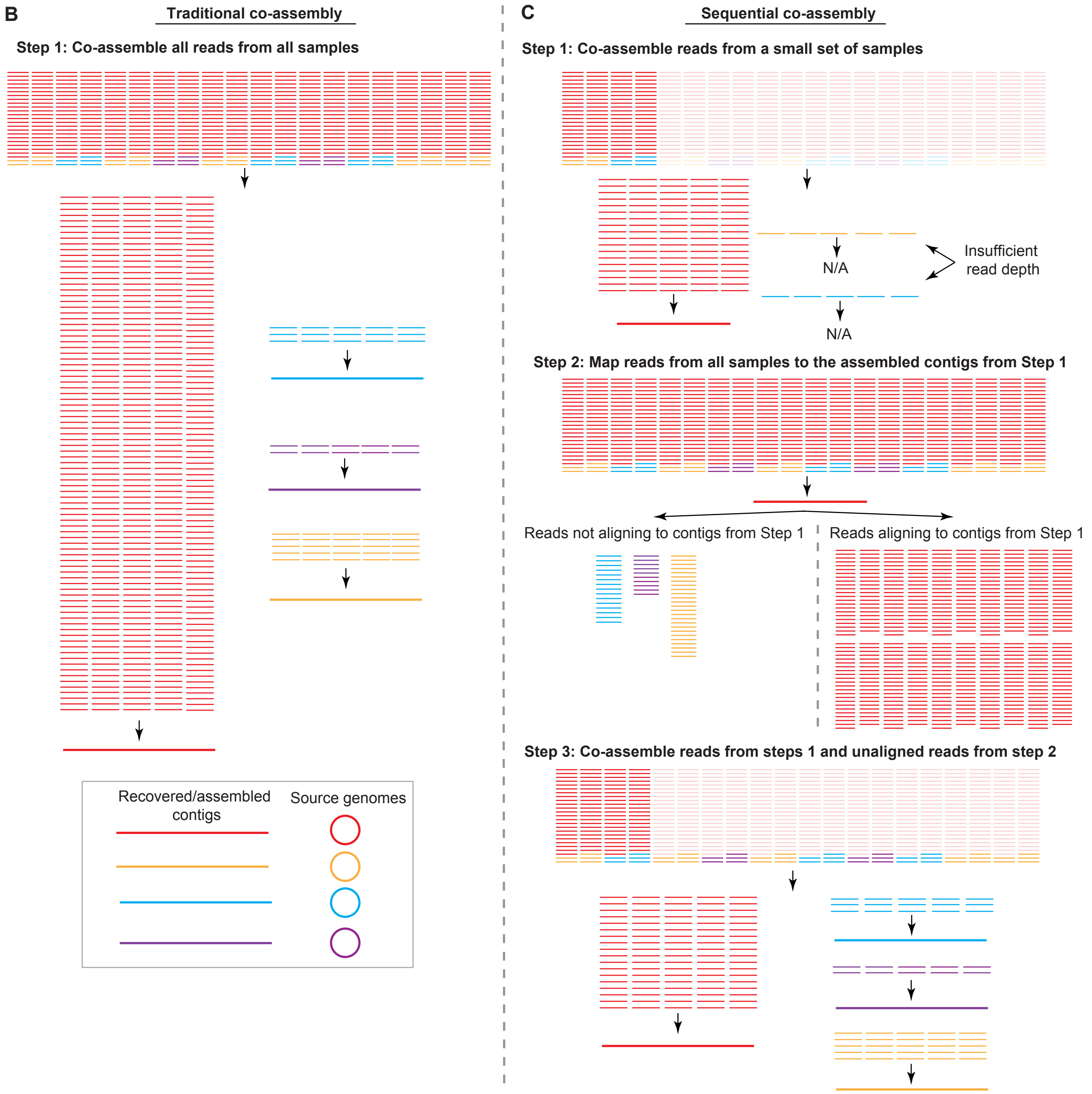

Figure S1: Comparison of traditional and sequential co-assembly (Related to STAR Methods). (A) Example dataset. There are 10 samples with differing relative abundances of four organisms and corresponding representative shotgun sequencing datasets. Each thin line represents a sequencing read. The color of the read matches the genome from which it was derived. **(B,C)** Schematics comparing traditional and sequential co-assembly approaches. Each thick line represents assembled contigs derived from the genome of same corresponding color. **(B)** Schematic of a traditional co-assembly strategy that involves combining and co-assembling all reads from all 10 samples. **(C)** Schematic of a multi-step, sequential co-assembly approach consisting of three steps.
